# Transthoracic echocardiography: an accurate and precise method for estimating cardiac output in the critically ill patient

**DOI:** 10.1186/s13054-017-1737-7

**Published:** 2017-06-09

**Authors:** Pablo Mercado, Julien Maizel, Christophe Beyls, Dimitri Titeca-Beauport, Magalie Joris, Loay Kontar, Antoine Riviere, Olivier Bonef, Thierry Soupison, Christophe Tribouilloy, Bertrand de Cagny, Michel Slama

**Affiliations:** 1 Medical Intensive Care Unit and INSERM U1088, Amiens University Hospital, Amiens, France; 2 Medical–Surgical ICU, La Florida Dr. Eloisa Diaz Insunza Hospital, Santiago, Chile; 3 Medical–Surgical Intensive Care Unit, Abbeville General Hospital, Abbeville, France; 4 Emergency Department, Saint Quentin General Hospital, Saint Quentin, France; 5 Cardiology Department and INSERM U1088, Amiens University Hospital, Amiens, France; 6 Medical Intensive Care Unit, CHU Sud, F-80054 Amiens cedex 1, France

**Keywords:** Cardiac output monitoring, Pulmonary artery catheter, Transthoracic echocardiography, Intensive care

## Abstract

**Background:**

Cardiac output (CO) monitoring is a valuable tool for the diagnosis and management of critically ill patients. In the critical care setting, few studies have evaluated the level of agreement between CO estimated by transthoracic echocardiography (CO-TTE) and that measured by the reference method, pulmonary artery catheter (CO-PAC). The objective of the present study was to evaluate the precision and accuracy of CO-TTE relative to CO-PAC and the ability of transthoracic echocardiography to track variations in CO, in critically ill mechanically ventilated patients.

**Methods:**

Thirty-eight mechanically ventilated patients fitted with a PAC were included in a prospective observational study performed in a 16-bed university hospital ICU. CO-PAC was measured via intermittent thermodilution. Simultaneously, a second investigator used standard-view TTE to estimate CO-TTE as the product of stroke volume and the heart rate obtained during the measurement of the subaortic velocity time integral.

**Results:**

Sixty-four pairs of CO-PAC and CO-TTE measurements were compared. The two measurements were significantly correlated (*r* = 0.95; *p* < 0.0001). The median bias was 0.2 L/min, the limits of agreement (LOAs) were –1.3 and 1.8 L/min, and the percentage error was 25%. The precision was 8% for CO-PAC and 9% for CO-TTE. Twenty-six pairs of ΔCO measurements were compared. There was a significant correlation between ΔCO-PAC and ΔCO-TTE (*r* = 0.92; *p* < 0.0001). The median bias was –0.1 L/min and the LOAs were –1.3 and +1.2 L/min. With a 15% exclusion zone, the four-quadrant plot had a concordance rate of 94%. With a 0.5 L/min exclusion zone, the polar plot had a mean polar angle of 1.0° and a percentage error LOAs of –26.8 to 28.8°. The concordance rate was 100% between 30 and –30°. When using CO-TTE to detect an increase in ΔCO-PAC of more than 10%, the area under the receiving operating characteristic curve (95% CI) was 0.82 (0.62–0.94) (*p* < 0.001). A ΔCO-TTE of more than 8% yielded a sensitivity of 88% and specificity of 66% for detecting a ΔCO-PAC of more than 10%.

**Conclusion:**

In critically ill mechanically ventilated patients, CO-TTE is an accurate and precise method for estimating CO. Furthermore, CO-TTE can accurately track variations in CO.

## Background

Cardiac output (CO) monitoring is a valuable tool for the diagnosis and management of critically ill patients. For decades, the standard technique for CO monitoring in the intensive care unit (ICU) has been based on intermittent thermodilution (ITD) measurements with a pulmonary artery catheter (PAC).

Transthoracic echocardiography (TTE) is a non-invasive means of hemodynamic assessment that can be applied to critically ill patients. TTE can be used to estimate CO in several ways. The most frequently recommended method involves measuring the blood flow velocity (using a Doppler technique) at the left ventricular outflow tract (LVOT) and thus obtaining the stroke volume (SV). Echocardiography is now recommended as the first evaluation of the patient in circulatory failure [1–3]. It is therefore very important to determine the reliability of TTE for the measurement of CO and variations in CO (ΔCO).

In the critical care setting, few studies have evaluated the level of concordance between CO estimated by TTE (CO-TTE) and CO estimated by PAC (CO-PAC) [4–6]. In these studies, Bland–Altman analyses have evidenced a small level of bias and a broad limit of agreement (LOA). None of these studies has calculated the precision of each technique and the ability of TTE to track ΔCO in critically ill patients has not been evaluated. Furthermore, there is no consensus on the best method of evaluating trending ability. Recently, a new statistical approach (polar plot analysis) has been applied. The polar plot’s main advantage is its ability to assess the direction and magnitude of ΔCO [7]. However, this new approach has not previously been used to compare CO-TTE and CO-PAC.

Hence, we decided to prospectively evaluate the precision of (and the level of agreement between) CO-TTE and the reference method CO-PAC. We also used a polar plot to evaluate the ability of TTE to track ΔCO (as measured with a PAC) in critically ill, mechanically ventilated patients.

## Methods

### The study population

This prospective, observational study was performed between January 2015 and May 2016 in a 16-bed ICU in a university hospital (Amiens, France). The investigational protocol was approved by the local independent ethics committee (CPP Nord Ouest II, Amiens, France). In line with French legislation, all patients (or their surrogates) were provided with study information and gave their informed consent to participate. We included all mechanically ventilated patients hospitalized in our ICU and fitted with a PAC due to respiratory or hemodynamic failure. Insertion of the PAC was decided by the attending physician in line with the practice in our ICU.

The main exclusion criteria were age under 18, arrhythmia, severe valvulopathy, severe tricuspid regurgitation, and poor echogenicity. All patients were on continuous IV sedation and were temporarily paralyzed during CO-PAC and CO-TTE measurement. The sedation level was monitored and adjusted according to our protocol by the attending medical staff. Both types of measurement were performed simultaneously by two different investigators. In order to assess the trending ability of TTE for ΔCO, a second set of CO measurements was recorded 24 hours later in a randomly selected subgroup of 23 patients. The following data were also recorded: age, gender, Simplified Acute Physiology Score (SAPS II), the main reason for admission to the ICU, and presence or absence of catecholamine infusion at the time of the CO measurements.

### Pulmonary artery catheter monitoring

A 6.0-F PAC (Swan-Ganz Thermodilution Pulmonary Artery Catheter 131HF7; Edwards Lifesciences, Irvine, CA, USA) was used for the CO measurements. All patients underwent a chest X-ray scan after insertion of the PAC to check for the correct position and the absence of complication. Also, to verify that the tip of the catheter was in the third zone of the West classification we used the methodology described by Teboul et al. [8] using the pressure wave forms. CO-PAC ITD measurements were made by injecting 10 ml of cold saline through the proximal port. Five consecutive injections were made randomly during the respiratory cycle. The five measurements were averaged to obtain the CO-PAC value. Arterial blood pressure was measured invasively in all patients. All patients were monitored in the supine position, and the zero reference was the mid-axillary line.

### Transthoracic echocardiography

Standard echocardiographic views were acquired using a Vivid S6 echocardiograph (GE Medical Systems, Milwaukee, WI, USA). Cardiac output was calculated from the left ventricular outflow tract (LVOT) as described by Mclean et al. [5]. The diameter of the LVOT was taken to be the distance between the bases of the aortic valve cusp during systole, as seen from the long parasternal view. The LVOT area was calculated assuming a circular geometry. In order to reduce variability we used the average of three measures of LVOT diameter. The LVOT area was then calculated as the product radius squared:

$$ \mathrm{LVOT}\ \mathrm{area} = \left[{\left(\mathrm{LVOT}\ \mathrm{diameter}\ \mathrm{average}\ /\ 2\right)}^2\right] \times 3.14 $$

Pulsated wave Doppler samples were then obtained in the center of the LVOT from the apical view, paying close attention to obtaining an angle of Doppler signal to aortic blood flow close to 0°. This condition is very important because Doppler ultrasonography is extremely dependent on alignment with the aortic blood flow and catching the signal at the proper angle (<20°) is fundamental to obtaining an adequate measure. The leading edge of five consecutive Doppler velocity curves was traced and the average velocity time integral (VTI) was calculated. The SV obtained with TTE (SV-TTE) was calculated as the product of the LVOT area and the VTI of the LVOT blood flow. The CO-TTE was calculated as the product of the SV-TTE and the heart rate obtained during measurement of the aortic VTI.

Left ventricular systolic function was assessed by the left ventricular ejection fraction, as measured with Simpson’s modified rule. TTE was performed at the same time as the PAC measurements by a highly experienced sonographer, who was blinded to the PAC measurements. All of the TTE measurements were acquired in accordance with the European Association of Cardiovascular Imaging/American Society of Echocardiography task force’s recommendations [9, 10] and then averaged over five consecutive cardiac cycles.

### Statistical analysis

The normality of the data distribution was checked using a Kolmogorov–Smirnov test. Data are reported as number (percentage) for categorical variables and median (interquartile range (IQR)) for continuous variables. The correlation between CO-PAC and CO-TTE measurements was quantified by calculating Pearson’s coefficient. Bland–Altman analysis was also used to evaluate the level of agreement between PAC and TTE. The percentage error (PE) was calculated. The precision of CO-PAC was calculated from five ITDs per patient. The precision of CO-TTE was calculated from five VTI consecutive measurements.

The changes in CO-PAC (ΔCO-PAC) and CO-TTE (ΔCO-TTE) were calculated by subtracting the first measurement from the second measurement. The correlation between ΔCO-PAC and ΔCO-TTE was quantified by calculating Pearson’s coefficient. Bland–Altman analysis was also used to evaluate the level of agreement between ΔCO-PAC and ΔCO-TTE. To evaluate the ability of TTE to track ΔCO-PAC, we performed a four-quadrant plot and a polar plot analysis. For the four-quadrant plot, we used the percentage changes in ΔCO and an exclusion zone of 15%. Good trending ability was defined as a concordance rate of more than 90% [11]. In the polar plot, we analyzed the absolute ΔCO changes and applied an exclusion zone of 0.5 L/min. Good trending ability was defined as a mean polar angle of less than ±5° and a radial LOA of less than ±30° [11, 12].

Furthermore, the ability of TTE to predict a ΔCO-PAC of more than 10% was analyzed in a receiver operating characteristic (ROC) curve analysis. The least significant change in the CO-PAC was also calculated.

All statistical analyses were performed with MedCalc software (version 12.0.4.0; MedCalc Software, Mariakerke, Belgium) and SigmaPlot software (version 11.0; Systat Software, San Jose, CA, USA). The threshold for statistical significance was set to *p* < 0.05.

## Results

### Patient characteristics

**Fig. 1:**
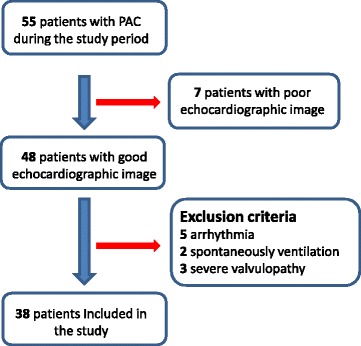
Study flow chart. *PAC* pulmonary artery catheter

A total of 38 sedated, mechanically ventilated patients were included (Fig. 1). Twenty-six (68%) of the patients were male, the median (IQR) age was 65 years (58–74), and the median (IQR) SAPS II was 67 (51–78). The most frequent cause for ICU admission was pneumonia (*n* = 24, 63%). The demographic characteristics of the study population are summarized in Table 1. A total of 64 pairs of CO measurements were recorded. There were only four patients with a RV/LV ratio > 1 in our population. The hemodynamic, respiratory, and echocardiographic data are also summarized in Table 1.

**Table 1:** Characteristics of the study population

Characteristic	Study population (*n* = 38)
Male	26 (68)
Age (years)	65 (58–74)
SAPS II	67 (51–78)
Admission	
Septic shock/SIRS	19 (50)
Cardiogenic shock	5 (13)
Hypovolemic shock	3 (8)
Respiratory failure	11 (29)
Indication for PAC monitoring	
Hemodynamic failure	24 (63)
Respiratory failure	14 (37)
Catecholamines	
Norepinephrine	28 (74)
Dobutamine	3 (7)
Epinephrine	2 (5)
Surgery	2 (5)
MAP (mmHg)	72 (67–85)
HR (bpm)	102 (81–119)
Sat O_2_ (%)	96 (93–98)
PaO_2_/FiO_2_ ratio	216 (133–294)
Mechanical ventilation	
Tidal volume (ml)	460 (373–500)
PEEP (cmH_2_O)	8 (5–10)
RR (cycle/min)	22 (19–26)
PPlat (cmH_2_O)	20 (17–23)
FIO_2_ (%)	45 (35–70)
LVEF (%)	58 (42–67)
CO-PAC (L/min)	5.8 (4.7–7.5)
CO-TTE (L/min)	5.8 (4.7–7.1)
SV-PAC (ml)	63 (46–78)
SV-TTE (ml)	64 (49–71)
RV/LV ratio	0.6 (0.6–0.7)
TAPSE (mm)	1.9 (1.6–2.3)

Data expressed as median (IQR) or *n* (%)

*SAPS II* Simplified Acute Physiology Score, *SIRS* systemic inflammatory response syndrome, *PAC* pulmonary artery catheter, *MAP* mean arterial pressure, *HR* heart rate, *Sat O*_*2*_ oxygen saturation, *PEEP* positive end-expiratory pressure, *RR* respiratory rate, *PPlat* plateau pressure, *FIO*_*2*_ fraction of inspired oxygen, *LVEF* left ventricular ejection fraction, *CO-PAC* cardiac output measured with a pulmonary artery catheter, *CO-TTE* cardiac output estimated by transthoracic echocardiography, *SV-PAC* stroke volume measured by pulmonary artery catheter, *SV-TTE* stroke volume estimated by transthoracic echocardiography, *TAPSE* tricuspid annular plane systolic excursion, *RV/LV ratio* ratio between right ventricle area and left ventricle area *PaO*_*2*_ arterial oxygen partial pressure *FIO*_*2*_ fractional inspired oxygen

### CO measurements

**Fig. 2:**
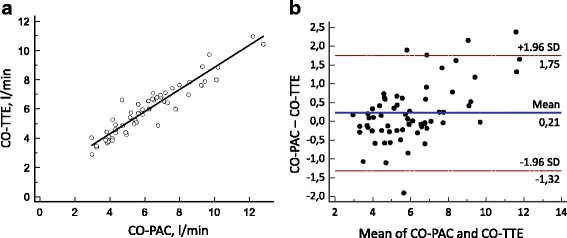
**a** Correlation between CO-PAC and CO-TTE (*r* = 0.95; *p* < 0.0001). **b** Bland–Altman plot for CO-PAC and CO-TTE (*n* = 64 pairs of measurements). *Solid line*: bias; *dashed line*: LOA. *CO-PAC* cardiac output measured by pulmonary artery catheter, *CO-TTE* cardiac output estimated by transthoracic echocardiography

Sixty-four measurements of CO-PAC and CO-TTE were compared. There was a significant correlation between the CO-PAC and CO-TTE measurements (*r* = 0.95; *p* <  0.0001; Fig. 2). The Bland–Altman analysis evidenced a median bias of 0.2 L/min and LOAs of –1.3 and 1.8 L/min (Fig. 2). The PE was 25%. The precision of CO measurement was 8% with the PAC and 9% with TTE.

### Variations in CO

**Fig. 3:**
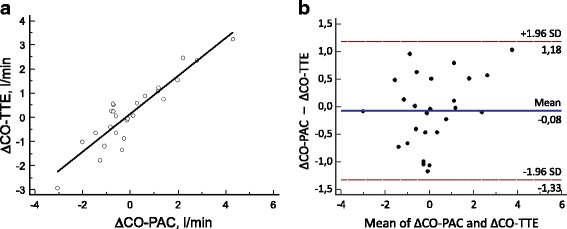
**a** Correlation between absolute values of ΔCO-PAC and ΔCO-TTE (*r* = 0.92; *p* < 0.0001). **b** Bland–Altman plot for ΔCO-PAC and ΔCO-TTE (*n* = 26 pairs of measurements). *Solid line*: bias; *dashed line*: LOA. *CO-PAC* cardiac output measured by pulmonary artery catheter, *CO-TTE* cardiac output estimated by transthoracic echocardiography

**Fig. 4:**
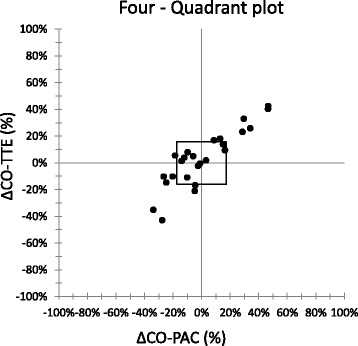
Four-quadrant plot of %ΔCO-TTE vs %ΔCO-PAC. A central exclusion zone of 15% (*square*) was applied. Concordance rate was 94%. *CO-PAC* cardiac output measured by pulmonary artery catheter, *CO-TTE* cardiac output estimated by transthoracic echocardiography

**Fig. 5:**
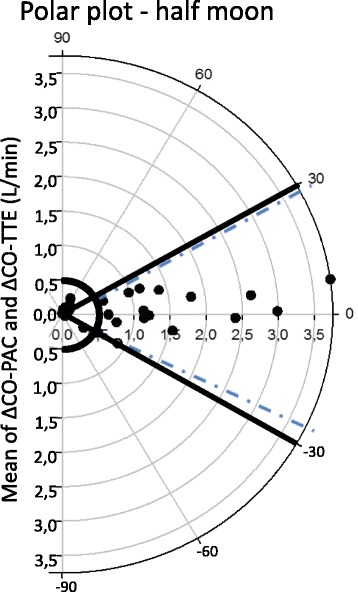
Polar plot showing changes in ΔCO-TTE in comparison with ΔCO-PAC. A central exclusion zone of 0.5 L/min (*half circle*) and ± 30° axes (*solid lines*) are indicated. Mean polar angle: 1.0°; 95% radial LOAs: –26.8 to 28.8° (*dotted lines*). Concordance rate (between –30 and 30°) was 100%. *CO-PAC* cardiac output measured by pulmonary artery catheter, *CO-TTE* cardiac output estimated by transthoracic echocardiography

Twenty-six pairs of ΔCO data were collected. There was a significant correlation between ΔCO-PAC and ΔCO-TTE (*r* = 0.92; *p* < 0.0001; Fig. 3). The Bland–Altman analysis evidenced a median bias of –0.1 L/min and LOAs of –1.3 and +1.2 L/min (Fig. 3). The four-quadrant plot had a concordance rate of 94% with a 15% exclusion zone (Fig. 4). With an exclusion zone of 0.5 L/min, the polar plot had a mean polar angle of 1.0° and LOAs of –26.8 to 28.8° (Fig. 5). The polar plot’s concordance rate was 100% between 30 and –30°. The area under the ROC curve (95% CI) for detecting an increase in ΔCO-PAC of more than 10% as a function of ΔCO-TTE was 0.82 (0.62–0.94) (*p* < 0.001). By monitoring a ΔCO-TTE of more than 8%, a ΔCO-PAC of more than 10% could be detected with a sensitivity of 88% and specificity of 67%.

## Discussion

In the present study of mechanically ventilated, critically ill patients, our comparative analysis of simultaneously measured CO-TTE and CO-PAC revealed a small level of bias and moderate LOA. Nevertheless, both techniques had an acceptable PE for CO measurements and very good trending ability for ΔCO. Furthermore, the CO-TTE measurements were found to be very precise. We conclude that TTE is an accurate and precise method for estimating CO in the critically ill patient.

More than 30 years ago, a number of echocardiography studies focused on patients having undergone cardiac surgery and, in particular, patients in the ICU [13–20]. These studies measured the CO by monitoring the Doppler signal at the aortic, pulmonary, or mitral valves. The data were compared by applying thermodilution, dye dilution, or Fick’s method. Other researchers calculated CO from the systolic and diastolic volumes of the left ventricle [21]. The earliest studies used pulsed or continuous wave Doppler techniques to measure the blood flow in a suprasternal view of the ascending aorta [15]. The best correlation with reference methods was found when the aortic blood flow was recorded from a pulsed Doppler apical five-chamber view of a sample volume located at the aortic annulus [18, 19]. The correlations were also good when blood flow was measured at the mitral valve, although this method was too complex for use in the ICU. Measurements of the left ventricular volume and the blood flow at the pulmonary valve were only weakly correlated with the reference method [21]. More recently, transesophageal echocardiography with thermodilution was found to be well correlated with the reference method for the assessment of CO in ICU patients [21–23]. Darmon et al. [24] assessed the capability of transesophageal echocardiography to determine CO in a transgastric long axis by comparison with CO measured by TD. In this study the authors assumed a triangular shape for the aortic valve orifice. The aortic valve orifice area was calculated as the area of an equilateral triangle. The authors showed very good correlation and Bland–Altman analysis in comparison with CO measured by TD [24]. In their study, however, the authors used TEE to evaluate directly the area of the aortic valve orifice, which is much harder using TTE as we used in our research and the Doppler technique using the LVOT measurement is the recommended technique in TTE to determine the CO.

Most of these early studies used linear regression to compare the Doppler and reference methods. However, it has recently been demonstrated that the accuracy and precision of these methods cannot be reliably determined on the basis of a correlation coefficient alone [11, 12, 25].

In line with all of the literature data, we observed a strong, statistically significant correlation between CO-PAC and CO-TTE [13–20]. Bland–Altman analysis showed a small degree of bias and moderate LOAs, which were very similar to those reported by McLean et al. [5] (bias: 0.2 L/min; 95% LOAs: –1.5 to +1.9 L/min). In a study of 41 patients having undergone a liver transplant, Marcelino et al. [6] found a small bias (–0.6 L/min) and LOAs (–1.8 and +0.6 L/min). Neither of these two studies calculated the PE or the precision. The clinical interpretation of the LOA is complex; although a LOA of ± 1.5 L/min is acceptable for septic shock patients with high CO, it would be unacceptable for patients with cardiogenic shock and low CO. Critchley and Critchley [25] have suggested that this problem can be resolved by using the PE of the LOA (relative to the mean value for the patient population) of ± 30% as a cutoff point. In our study, the PE was 25%.

We found that TTE was able to track ΔCO-PAC with a good concordance rate of 94.4%, according to a four-quadrant plot with an exclusion zone of 15% (as recommended by Critchley et al. [12]). Only one pair of ΔCO-TTE measurements changed in a different direction. Given that the four-quadrant plot only analyzes the inter-method agreement in the direction of ΔCO, we used the recently described polar plot to analyze trending ability. The polar plot allows analysis of the direction and magnitude of ΔCO. We observed an angular bias of 1.0°, LOAs of –26.8 to 28.8°, and an excellent concordance rate of 100% below ±30°. In the polar plot of absolute ΔCO changes, we applied an exclusion zone of 0.5 L/min [11]. A mean polar angle of less than ±5° and a radial LOA of less than ±30° correspond to good trending ability. Concordance rates above 90% are also considered to reflect good trending ability [11]. In both analyses, 17 of the 26 pairs of ΔCO measurements fell outside the exclusion zone and were therefore included in the analysis. Lastly, the ROC curve showed that a ΔCO-TTE > 8% is able to identify a variation of CO-PAC > 10% with a sensitivity of 88% and a specificity of 66%. We tested a ΔCO-PAC of 10% because the least significant change in the CO-PAC was 9% in our study and because a ΔCO greater than 10% is usually considered as the cutoff point for a hemodynamic response to volume expansion in clinical practice.

In the ICU, variations in CO are monitored as a guide to the effects of volume expansion and catecholamine drugs. The very good trending ability displayed by TTE in the present study suggests that this technique is a good means of assessing variations in CO. The present study is the first to have used a polar plot to evaluate the trending ability of TTE in critically ill patients.

TTE has some significant advantages over the PAC, and these should be taken into account in clinical validation studies. Firstly, TTE is a simple, non-invasive method that is as almost as precise as the reference method. Secondly, it has a smaller PE than the other non-invasive or minimally invasive devices currently in use.

Over recent decades, several CO measurement devices have been developed and then compared with the PAC [26–28]. The FloTrac/Vigileo is a non-invasive device that estimates CO from the arterial pressure waveform. Monnet et al. [29] reported a PE of 54% when compared with a third-generation FloTrac/Vigileo and CO-PAC. Furthermore, the FloTrac/Vigileo did not track ΔCO very accurately (with concordance rates of 73 and 60% in a four-quadrant plot). In a comparison of transesophageal echocardiography and PAC for the estimation of CO, Møller-Sørensen et al. [30] reported a PE of 38% and a precision of 16%. The latter researchers also observed poor ΔCO tracking ability, with a small angular bias (2.8°) but poor radial LOA (±53°) on the polar plot. Ostergaard et al. [31] compared CO-PAC with CO measured by transpulmonary thermodilution (TP-TD) and the pulse contour method (PiCCOplus) in patients before cardiac surgery; the PE was 21.2% for TP-TD vs CO-PAC and 50% for PiCCOplus vs CO-PAC.

We performed the measurement of CO-PAC and CO-TTE under optimal conditions; the patients were sedated and temporarily paralyzed, which decreased the level of patient–ventilator interaction. However, the present study had several limitations. The study population was small, although we were able to make CO measurements in patients with cardiogenic shock and patients with distributive shock.

We did not include patients with arrhythmia, in whom SV measurement is associated with large beat-to-beat variations. In the future, it would be interesting to focus on this subgroup of ICU patients.

Another limit of our study is that we tracked the CO changes after a period of 24 hours. We could have recorded the CO after fluid administration or any other intervention. This study was observational and in our practice when patients have a PAC the attending physician most of the time uses the PAC as the monitoring tool rather than echocardiography. However, echocardiography is performed at least once a day to check for the right and left ventricular functions, the development of an acute core pulmonale, or any morphologic abnormalities that could not be detected by the PAC. This is the reason why in this observational study the CO changes were analyzed after 24 hours.

## Conclusions

In a population of non-arrhythmic, mechanically ventilated ICU patients, we demonstrated that TTE is an accurate and precise non-invasive technique for evaluating and tracking changes in CO.

## Abbreviations

CO: Cardiac output
CO-PAC: Cardiac output measured by pulmonary artery catheter
CO-TTE: Cardiac output estimated by transthoracic echocardiography
ICU: Intensive care unit
ITD: Intermittent thermodilution
LOA: Limit of agreement
LVOT: Left ventricular outflow tract
PAC: Pulmonary artery catheter
SV: Stroke volume
SV-TTE: Stroke volume estimated by transthoracic echocardiography
TTE: Transthoracic echocardiography
ΔCO: Cardiac output variations
